# A Survey to Determine the Zone of Equipoise for the Proximal FEmur Resection or Internal Fixation fOR Metastases (PERFORM) Randomized Controlled Trial

**DOI:** 10.21203/rs.3.rs-4810027/v1

**Published:** 2024-10-16

**Authors:** Jessa Fogel, Vincent Ng, Thomas Schubert, Jonathan Forsberg, R. Lor Randall, Ricardo Becker, Carol Morris, Michelle Ghert

**Affiliations:** University of Maryland Medical Center; University of Maryland Medical Center; Université catholique de Louvain: Universite Catholique de Louvain; Memorial Sloan Kettering Cancer Center; UC Davis Health; Hospital de Clinicas de Porto Alegre; Memorial Sloan-Kettering Cancer Center Inpatient Hospital and Main Campus: Memorial Sloan Kettering Cancer Center; University of Maryland Medical Center

**Keywords:** survey, PERFORM, proximal femur, metastatic bone disease, orthopedic oncology

## Abstract

**Objective:**

The objective of this study was to establish a zone of clinical equipoise for the **P**roximal F**E**mur **R**esection or Internal **F**ixation f**OR M**etastases (PERFORM) randomized controlled trial, which will compare resection and endoprosthetic reconstruction to internal fixation for skeletal metastases of the proximal femur.

**Methods:**

A survey was developed, piloted, and distributed to self-declared interested stakeholders in the PERFORM trial. The survey targeted orthopaedic oncologists and was designed to assess patient and bone lesion characteristics that drive surgical decision making in the treatment of skeletal metastases in the proximal femur. An Ethics Waiver was obtained at the lead academic institution and data was collected in the REDCap survey database.

**Results:**

Responses were complete from 76 surgeons across North America, South America, Europe, Asia and Africa. Responses indicate that a study population for which either resection and endoprosthetic reconstruction or internal fixation are acceptable options include: (1) life expectancy at least 6 months, (2) bone loss of no more than 75% and no less than 25%, and (3) minimal to moderate risk for perioperative complications. Ninety-three percent of respondents indicated that they would be interested in participating in the PERFORM trial.

**Conclusion:**

A preliminary zone of equipoise for the PERFORM trial includes patients with 25–75% bone loss, low to moderate risk of operative complications, and life expectancy of at least 6 months. Further stakeholder discussions will finalize the PERFORM trial protocol prior to study initiation.

## Background

Metastatic bone disease presents significant morbidity to patients and cost to the healthcare system, accounting for an estimated 17% of cancer-related cost burden in the United States.^1^ The proximal femur is one of the most common sites of skeletal metastases in cancer patients, and as a weight bearing region subjected to high biomechanical forces, it is at elevated risk of fracture. It is estimated that pathologic fractures occur in 10–30% of cancer patients, a significant number of which occur in the proximal femur, resulting in substantial morbidity^2; 3^. Approximately 50% of metastatic proximal femoral lesions occur in the intertrochanteric or subtrochanteric region, with the other 50% located in the femoral neck.^4; 5^

Currently, the mainstay of treatment for proximal femur bone metastases that involve the intertrochanteric or subtrochanteric region is prophylactic surgical internal fixation, most commonly with intramedullary nailing. This has a number of advantages, including protection of the entire bone, minimal dissection of soft tissue, low cost, simplicity of procedure, and low infection rate.^6; 7^ However, prophylactic fixation carries the risk of disease progression and/or progression and subsequent implant failure.^8; 9; 10; 11^

Alternatively, an increasing number of surgeons are using endoprosthetic resection and reconstruction as a surgical technique to address metastatic bone disease of the proximal femur, particularly in patients with extensive intertrochanteric and subtrochanteric bone loss.^4; 12^ This carries the advantage of immediate stability without the need for bone healing, as well as resection of all local disease, reducing risk of local recurrence. However, these procedures are more complex and costly, involve larger areas of soft tissue dissection and greater blood loss, and carry their own set of complications, such as periprosthetic infection and hip dislocation, making this procedure less appropriate for palliative surgery^10; 11; 13; 14^.

Historically, the median survival for patients with bone metastases has been relatively short but varies by cancer primary diagnosis. However, with advancing treatments, patients may now live years after their cancer diagnosis.^2; 3^ Thus, an increasing percentage of patients who would historically be treated with an intramedullary nail may be more appropriately treated with resection and reconstruction. A study by the Royal College of Surgeons estimated a nail breakage rate of up to 16% in patients with pathologic fractures, with a mean time to breakage of 10 months^15^. Longer life expectancy in cancer patients with advances in oncologic treatment may lead to a greater number of patients “outliving” their intramedullary fixation.

The **P**roximal F**E**mur **R**esection or Internal **F**ixation f**OR M**etastases (PERFORM) study is a proposed randomized control trial that aims to compare patient-centered outcomes following resection and endoprosthetic reconstruction with internal fixation in patients with metastatic bone disease of the proximal femur. In order to design a valid study protocol, a study population needs to be defined in order to establish a zone of clinical equipoise between the two proposed treatment arms. The aim of the current study was to survey orthopedic oncologists regarding their practices in the treatment of proximal femur bone metastases in order to understand which patients may be appropriate for randomization.

## Methods

### Question development

The survey was developed using relevant concepts and approaches identified in the literature.^16; 17^ An initial draft of the survey was reviewed by the senior authors and revised for face and content validity. The survey was then piloted on a group of international orthopaedic oncology surgeons who provided feedback on the validity of the content and the logical flow of the survey. A final revised version was created on REDCap.

### Survey format and content

The questionnaire consisted primarily of closed-ended questions as multiple choice or numerical rank format. Several opportunities for open-ended feedback were included to provide additional insight into future trial design and patient selection.

The first portion of the survey included participant demographics. The second portion of the survey asked participants to rank a list of 8 patient characteristics or lesion characteristics in order of importance when deciding on surgical treatment, including age of patient, life expectancy, baseline ambulatory status, degree of bone destruction, medical comorbidities, impending vs pathologic fracture, number of other bone metastases, and number of visceral metastases.

Participants were then asked to determine if certain patient or lesion characteristics would lead them to be more likely to perform internal fixation, resection and endoprosthetic reconstruction, either technique, or report if the characteristic was not important in their decision-making process. These characteristics included 1) patient factors (age, life expectancy, comorbidities, ambulatory status); 2) disease specific factors (number of visceral and bone metastases); and 3) anatomic factors (lytic vs blastic lesion, location of lesion, degree of bone destruction, impending vs pathologic fracture, and lesion extent to soft tissue)

Additionally, participants were asked whether they conceptualized standard arthroplasty (i.e., all arthroplasty options) as more consistent with internal fixation or resection and endoprosthetic reconstruction, as well as whether they would be willing to participate in the PERFORM trial. Participants were also provided the opportunity to include any comments or feedback regarding the proposed PERFORM trial. The survey is available in Supplementary Material.

### Survey distribution:

Prior to survey distribution, an Ethics Waiver was obtained at the lead academic institution. The survey was then distributed by email to approximately 60 self-declared interested stakeholders in the PERFORM trial, who were initially contacted through the collaborative trial group previously established by the senior author – many who are members of the Musculoskeletal Tumor Society, as well as to the listserv of the European Musculoskeletal Oncology Society (approximately 300 orthopaedic oncology surgeons). Data was collected in the REDCap survey database and all responses were completely anonymous. Consent for the study was inferred by survey completion.

### Data analysis

Charts and tables of descriptive data were generated by the REDCap database. Analysis of results was based on equipoise ranges as discussed by Araki et al., in which situations where the majority of participants (greater than 50%) reported they would use either technique was considered a zone of equipoise.^16^

## Results

### Demographics of respondents:

A total of 76 responses were collected from surgeons across North America, South America, Europe, Asia and Africa representing a response rate of approximately 22%. The average age of respondents was 48 years, with 87% identifying as male. A wide range of practice experience was represented, with 18% of respondents having less than 5 years of practice experience, 27% with 5–10 years, 27% with 11–15 years, 24% with 16–20 years, and 3% with over 20 years of practice experience. The majority of respondents completed a musculoskeletal oncology fellowship and/or specialized training in musculoskeletal oncology, and work at an academic medical center. The vast majority of respondents (94%) had a practice consisting of greater than 50% bone and soft tissue tumors. Similarly, a vast majority (97%) supervise residents in training.

### Highly ranked factors

**Figure 1:**
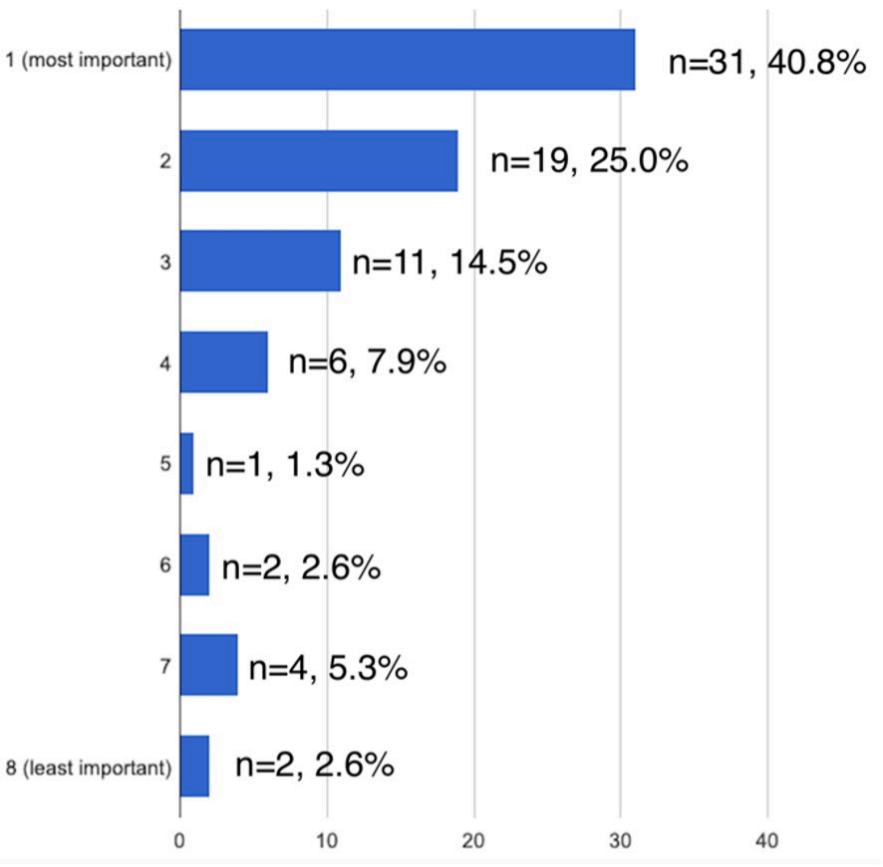
Importance of life expectancy in operative decision making

**Figure 2:**
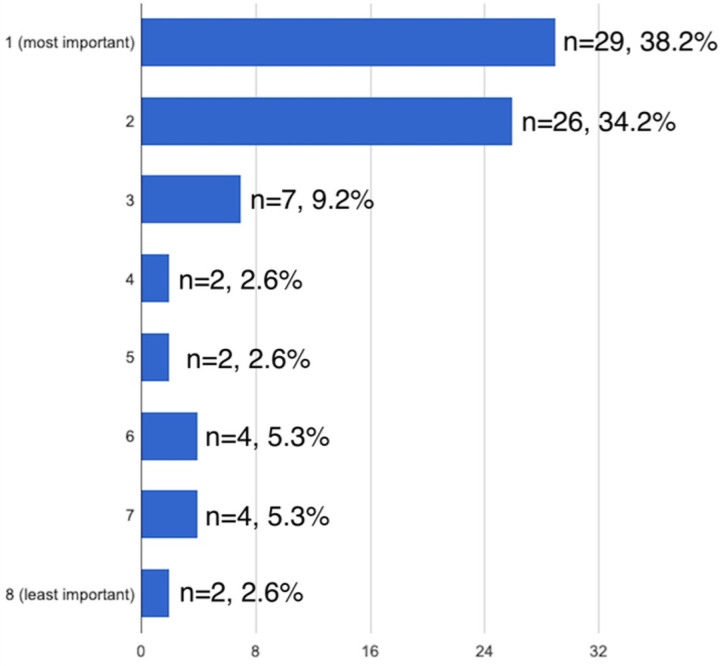
Importance of degree of bone destruction in operative decision making

The overall most important ranked factors in surgical decision making were life expectancy of patient (40.8% ranked as #1 most important, Fig. 1) and degree of bone destruction (38.2% ranked as #1 most important, Fig. 2). Baseline ambulatory status, medical comorbidities, and impending vs pathologic fracture were also consistently ranked as important factors.

### Patient factor zone of equipoise

Patient specific factors falling within the zone of equipoise (greater than 50% of respondents answering that they would use either technique) included life expectancy greater than 6 months, minimal to moderate perioperative complication risk, and patients of any age. Respondents were more likely to choose internal fixation for patients with less than 6 months of life expectancy and high perioperative complication risk.

### Disease factor zone of equipoise

Disease specific factors falling within the zone of equipoise included 2 or more bone metastases and up to 3 visceral metastases. Respondents were more likely to choose resection and endoprosthetic reconstruction for patients with solitary bone metastasis, and more likely to choose internal fixation for patients with greater than 3 visceral metastases.

### Anatomic zone of equipoise

**Figure 3:**
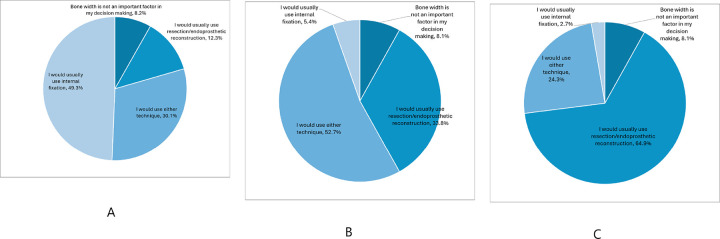
A: Responses for bone loss <25% B: Responses for bone loss 25–75% C: Responses for bone loss >75%

Anatomic factors falling within the zone of equipoise included both intertrochanteric and subtrochanteric lesions; lytic, blastic, or mixed lesions; lesions that extended or did not extend to soft tissue; impending and pathologic fractures (either displaced or nondisplaced); and bone loss of 25–75% (Fig. 3B). Respondents were more likely to choose resection and endoprosthetic reconstruction for patients with < 25% bone remaining (Fig. 3C). However, they were more likely to choose internal fixation for patients with > 75% bone remaining (Fig. 3A).

### Miscellaneous

In general, participants were slightly more likely to conceptualize standard arthroplasty as more consistent with resection and endoprosthetic reconstruction (n = 39, 52.0%). In response to what factors would lead respondents to consider internal fixation over resection and endoprosthetic reconstruction in the setting of bone metastases to the proximal femur in renal cell carcinoma, the most common responses were life expectancy (81.8%), extent of medical comorbidities (51.5%), and number of visceral metastases (30.3%). Based on the details of the trial outlined in the introduction of the survey, 93% of respondents indicated that they would be interested in participating in the PERFORM trial.

## Discussion

### Summary of Findings

The process of designing a randomized control study to compare two different surgical techniques is a highly challenging task, requiring careful planning and patient selection, as well as dedicated stakeholder cooperation. This survey aimed to identify patient and lesion characteristics that would fall into a zone of equipoise whereby most surgeons would be equally likely to choose internal fixation or resection and endoprosthetic reconstruction to treat a patient with metastatic bone disease of the proximal femur. This survey study found that a preliminary zone of equipoise would include patients with at least 6 months life expectancy, 25–75% bone loss in the proximal femur, and patients with low to moderate risk of perioperative complications.

### Implications for the PERFORM Trial

Given the diverse group of surgeons surveyed, a free text portion of the survey was included for feedback and comments regarding the trial. While many respondents used this section to convey their excitement and support for the trial, a few expressed concerns over the difficulty of randomization. Several respondents also noted that they felt that the type of cancer was an important factor in their decision making. However, the implications of ‘cancer type’, such as life expectancy and character of the lesion, were carefully assessed in the survey. Finally, while this was not a specific question included on the survey, renal cell carcinoma with oligometastatic bone disease is an exclusion factor for PERFORM trial patients, as the standard of care in the vast majority of cases is complete resection of the lesion, and therefore this scenario does not lie within the zone of equipoise.

In regards to the conceptualization of standard arthroplasty as more consistent with resection and endoprosthetic reconstruction vs internal fixation, there were a variety of opinions expressed. While a slight majority of respondents did consider arthroplasty as more consistent with resection and endoprosthetic reconstruction, a significant number (n = 23, 30.7%) did not consider it to be consistent with either technique. Those who felt arthroplasty was more consistent with resection and reconstruction cited reasons such as larger incision and soft tissue dissection, the resection of bone for standard arthroplasty, the use of implants for replacement rather than fixation, and no requirement for bone healing with arthroplasty. Those who felt arthroplasty was more consistent with internal fixation cited reasons such as arthroplasty being an intralesional resection without complete resection of the tumor, and the use of an arthroplasty stem as a means of internal fixation and stabilization. Given the lack of consensus on this point, and the fact that arthroplasty is not often the surgical approach of choice in intertrochanteric and subtrochanteric lesions, it is likely that the use of standard arthroplasty will be an exclusion criterion for the PERFORM trial.

### Strengths and Limitations

A recent modified Delphi study surveying both patients and orthopedic oncologists identified evaluation of surgical options for metastatic bone disease to be one of the top 3 research questions out of a list of nearly 200 options.^18^ There is clearly a clinical evidence gap, which the PERFORM study aims to fill. The current survey was a critical step in study design for the PERFORM trial. The survey was rigorously designed with an in-depth literature review and previous work by the investigators. The survey was iteratively assessed for face and content validity, and piloted to confirm ease of survey completion.

Although the response rate at 22% is relatively low, the vast majority of respondents indicated interest in participating in the PERFORM trial, and therefore represent the target audience of potential PERFORM trial investigators. In addition, respondents were from five continents and therefore provide generalizability of the survey data, as well as the identification of a large and diverse group of participating institutions. An inherent limitation in this study is the acknowledged challenge of selecting a true zone of equipoise in a heterogeneous patient population. Other limitations of this study include the potential for language or cultural barriers in question design, as well as the higher cost and variable availability of endoprostheses across institutions globally, which may have an impact on implant selection.

## Conclusion

The current survey successfully identified a clinically acceptable zone of equipoise for the PERFORM trial. The criteria for equipoise include patients with 25–75% bone loss in the proximal femur, low to moderate risk of operative complications, and life expectancy of at least 6 months. Ninety-three percent of respondents were willing to participate in a randomized control trial to evaluate surgical treatment options for metastatic bone disease of the proximal femur. Further stakeholder discussions will finalize the PERFORM trial protocol prior to study initiation.

## Data Availability

All data generated or analyzed during this study are included in this published article and its supplementary information files.
